# Temporal context effects are associated with cognitive status in advanced age

**DOI:** 10.1007/s00426-021-01502-9

**Published:** 2021-03-22

**Authors:** Sarah Maaß, Thomas Wolbers, Hedderik van Rijn, Martin Riemer

**Affiliations:** 1 Department of Experimental Psychology, University of Groningen, Grote Kruisstraat 2/1, 9712-TS Groningen, The Netherlands; 2 Behavioral and Cognitive Neurosciences, University of Groningen, Groningen, The Netherlands; 3 Aging and Cognition Research Group, German Center for Neurodegenerative Diseases (DZNE), Magdeburg, Germany; 4 Center for Behavioral Brain Sciences (CBBS), Magdeburg, Germany

## Abstract

The perception of temporal intervals changes during the life-span, and especially older adults demonstrate specific impairments of timing abilities. Recently, we demonstrated that timing performance and cognitive status are correlated in older adults, suggesting that timing tasks can serve as a behavioral marker for the development of dementia. Easy-to-administer and retest-capable timing tasks therefore have potential as diagnostic tools for tracking cognitive decline. However, before being tested in a clinical cohort study, a further validation and specification of the original findings is warranted. Here we introduce several modifications of the original task and investigated the effects of temporal context on time perception in older adults (> 65 years) with low versus high scores in the Montreal Cognitive Assessment survey (MoCA) and a test of memory functioning. In line with our previous work, we found that temporal context effects were more pronounced with increasing memory deficits, but also that these effects are stronger for realistic compared to abstract visual stimuli. Furthermore, we show that two distinct temporal contexts influence timing behavior in separate experimental blocks, as well as in a mixed block in which both contexts are presented together. These results replicate and extend our previous findings. They demonstrate the stability of the effect for different stimulus material and show that timing tasks can reveal valuable information about the cognitive status of older adults. In the future, these findings could serve as a basis for the development of a diagnostic tool for pathological cognitive decline at an early, pre-clinical stage.

## Introduction

The perception of time is of fundamental importance for a variety of cognitive and behavioral functions (e.g., motor planning, the timing of pauses in speech, or optimal preparation to a temporally predictable event), and it is therefore not surprising that timing deficits also play an important role in many neurodevelopmental and -degenerative disorders. Pathological distortions of time perception have been discussed with respect to Parkinson’s disease (Malapani et al., 1998; Mioni et al., 2018), depression (Mioni et al., 2016; Thönes & Oberfeld, 2015), attention deficit hyperactivity disorder (Kerns et al., 2001; Noreika et al., 2013), as a potential cause for circumscribed symptoms in schizophrenia (Giersch et al., 2015; Riemer, 2018), and have been proposed as a cognitive marker for the diagnosis of dementia (El Haj & Kapogiannis, 2016; Maaß et al., 2019).

As timing deficits might be related to impaired cognitive functioning in advanced age, we recently investigated the effect of temporal contexts on the performance in time reproduction tasks (Maaß et al., 2019). Participants were presented with a standard interval of a specific duration and were then asked to terminate another interval when it reached the same duration as the standard. After the presentation of several different standard intervals, a central tendency of responses was observed: The longer durations were under-reproduced, whereas the shorter durations were over-reproduced. We demonstrated that this context effect is significantly larger in patients suffering from amnestic mild cognitive impairment (aMCI), and the effect even scales with the scores on general memory tests in healthy older participants. This suggests that temporal context effects can be used as a behavioral marker for memory deficits and cognitive decline in a very early stage. Thus, when memory functions deteriorate, humans seem to be more strongly influenced by contextual information as their time judgments are more biased by expectations and most recent experiences than healthy controls (Maaß et al., 2019).

Short and long temporal contexts can be elicited in many ways (Bratzke & Bryce, 2016; Maaß et al., 2019), and are typically created by blocked presentation (e.g., one block of short intervals ranging from 1 to 3 s, followed by a separate block of long intervals ranging from 3 to 5 s). However, it is unknown to date whether distinct temporal contexts can also influence timing behavior in a mixed block in which both contexts are presented together and are indicated only by specific characteristics of the stimuli (e.g., short intervals are presented by stimuli on the left side and long intervals by stimuli on the right side of the screen, as in Roach et al., 2017). In this situation, temporal contexts are only indicated by the stimulus features (e.g., its location).

From a Bayesian perspective, the presence of context effects within a mixed block presumes the establishment and/or maintenance of two concurrent priors rather than establishing two contexts in a serial fashion as is the case in separate blocks. Roach et al. (2017) demonstrated that, in healthy young adults, a substantial number of experiences with the distinct contexts is needed to establish two temporal contexts within mixed blocks (see also Taatgen & van Rijn, 2011, who explored contamination between two contexts in a mixed block). As the maintenance of two distinct contexts within the same experimental block can be assumed to require enhanced cognitive effort (as compared to the maintenance of just one, e.g., Roach et al., 2017), it could be argued that cognitive deficits would impede context effects in a mixed block. However, given the increased susceptibility to contextual information in participants with cognitive deficits (Maaß et al., 2019), it is also possible that context effects in a mixed block are even larger in participants with first signs of cognitive decline, compared to an age-matched group without cognitive deficits. Testing these contrary assumptions is pertinent to determine the usefulness of temporal context effects for the diagnosis of cognitive decline in early stages.

Another important issue in the research on timing deficits is the reliance on artificial, abstract stimuli (Boltz, 2005; Matthews & Meck, 2014; van Rijn, 2018). Although the use of abstract stimuli (e.g., simple visual shapes) has a clear advantage in terms of experimental controllability, it often lacks ecological validity and the results might not be generalizable to real-world timing, and therefore the use of more realistic stimuli and paradigms has been proposed (Riemer et al., 2018; Thanopoulos et al., 2018; van Rijn, 2014). Time perception in the real world is influenced by the detection of regularities and temporal expectations (Tanaka & Yotsumoto, 2017), while these factors should be less important for the timing of abstract stimuli which have no inherent meaning and usually are not linked to specific durations or regularities.

An efficient strategy to cope with the perceptual complexity of the real world is to utilize environmental regularities in order to generalize from a few experiences to broader categories (Son et al., 2008), and there is evidence that this process occurs automatically without conscious control (Sutherland et al., 2015). Furthermore, it has been suggested that the processing of environmental contingencies depends on the realistic appearance of experimental stimuli (Peelen & Kastner, 2014; Thanopoulos et al., 2018). For example, Thanopoulos et al. (2018) found that the tendency to underestimate the interval between a self-initiated action and a subsequent visual stimulus (a phenomenon known as intentional binding; see Haggard et al., 2002) is more pronounced when the visual stimulus is a realistic image and semantically related to the action, than when it is an abstract shape. This shows that a realistic embedding of stimuli enhances the processing of their relation. Differential effects of realistic and abstract stimuli have also been reported in visual detection tasks, because regularities of visual content are expected for realistic but not abstract images (Peelen & Kastner, 2014).

As memory processes for durations have been shown to resemble those of visual content (Fan & Yotsumoto, 2018), a similar mechanism might also affect the processing of temporal regularities. For example, when we repeatedly estimate the duration of illuminated windows in a visual scene, the natural embedding of the durations might trigger a stronger inclination to categorize them according to the temporal regularities (i.e., if one window is, on average, illuminated for relatively long intervals). Assuming that naturalistic stimuli lead to a stronger categorization, such an influence on the processing of single durations would be greater when presented in a semantically complex visual environment in which temporal regularities are expected. This would result in stronger context effects for realistic scenes as compared to abstract stimuli. Although this should affect context effects independent of whether they are assessed in separate or mixed blocks, the influence of realistic stimuli might be relatively more pronounced for mixed-block context effects, because here the different contexts are presented during the same experimental block, rendering the differentiation between environmental aspects (to which the contexts are linked) more relevant (Roach et al., 2017).

Using abstract, acoustic stimuli (i.e., uniform sounds), we have shown increased context effects in a clinical population suffering from amnestic mild cognitive impairment (aMCI) compared to healthy age-matched controls, and within this latter group, context effects are stronger in individuals performing relatively low in a memory test compared to high-performing individuals (Maaß et al., 2019). Building upon these findings, we investigated here in two groups of older individuals with low versus high scores on tests of cognitive functioning, whether the effect of temporal contexts (learned within two separate blocks) can also be observed when tested in a subsequent mixed block, in which the two contexts are presented in a mixed manner.

We hypothesized (1) that context effects are larger in participants with cognitive deficits, (2) that they carry over to a finally presented mixed block containing both contexts, generalizing our earlier findings in which we tested only separate blocks, and (3) that, due to top-down influences, the impact of temporal contexts is increased for the timing of realistic stimuli as compared to the timing of abstract visual stimuli. A confirmation of the latter hypothesis will provide valuable information about the optimal conditions under which context effects can be assessed.

## Methods

### Participants

Forty participants (all above 65 years old) were recruited from the local community in Magdeburg, Germany, by means of public advertisements in local newspapers. All participants were tested individually at the University Hospital Magdeburg. Cognitive functioning, focusing on memory skills, was assessed with a delayed word recall task (identical to the word list recall task of the CERAD test battery, but with the default words replaced by words matching in complexity; Fillenbaum et al., 2008; Morris et al., 1989), and the Montreal Cognitive Assessment survey (MoCA, alternative version three; Nasreddine et al., 2005). Word recall scores ranged from 3 to 10, with a mean of 6.6 correct responses. However, to account for differences associated with education, age and sex, word recall scores were expressed as a distance to a threshold score that is based on a participant’s education, age and sex. Scores on this distance measure ranged from − 2.4 to 5.95, with a mean of 1.7 (8 participants below the cut-off). Based on an education-corrected MoCA cut-off criterion of 26 (Nasreddine et al., 2005), participants were assigned to a group with few or no cognitive deficits (11 females, 12 males; mean age 69.6 years, ranging from 65 to 75, MoCA score ≥ 26) and a group that failed this threshold (9 females, 8 males; mean age 69.5 years, ranging from 65 to 80, MoCA score < 26).

One participant from the group with few or no cognitive deficits completed only half of the experiment and was removed from further analyses. Participants received monetary compensation and gave written informed consent to the experimental protocol that was approved by the DZNE ethics committee.

### Task and stimuli

**Fig. 1:**
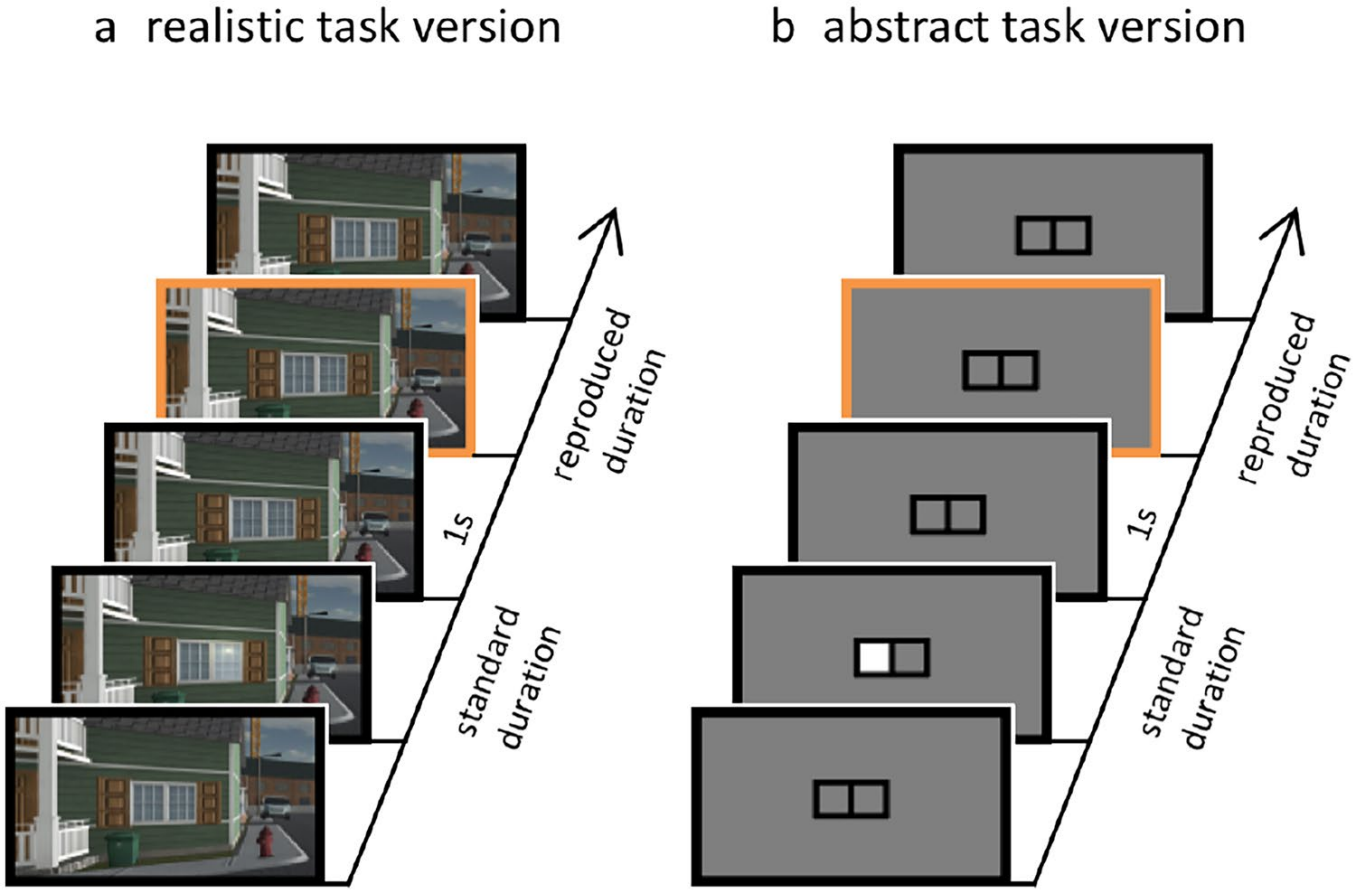
Outline of the experimental task. In each trial, participants reproduced the duration of **a** a lit window in the realistic version, and **b** a white square in the abstract version

Participants were seated in front of a computer monitor and saw a visual scene depicting the front of a house or an abstract frame on grey background (Fig. 1). In each trial, either the left or the right window (or left or right panel of the frame) was illuminated for a specific duration, and then turned dark again. Participants were asked to register the duration of the illumination. After an inter-stimulus interval of one second, an orange frame appeared around the screen and remained until the spacebar was pressed. Participants were instructed to press the spacebar as soon as the orange frame was presented for the same duration as the window was illuminated.

The experiment consisted of two parts (see Table 1), repeated twice, once using the realistic scene and once using the abstract stimuli, the order of which was counterbalanced across participants. In the first part, participants were either first presented with durations sampled from a short context (1066, 1600, and 2400 ms), followed by durations sampled from a long context (2400, 3600 and 5400 ms), or vice versa. The short and long contexts were associated either with the left or the right spatial side. Both context order and association with spatial side were counterbalanced across participants. In the second part, presented durations were randomly sampled from both contexts, yet each context was still associated with the same spatial side. The first part will be referred to as the *separate* context blocks, whereas the second part will be referred to as the *mixed* context block. Note that both contexts contain a medium standard duration of 2400 ms on which the analyses will focus. In the separate context blocks, each duration was presented 5 times for a total of 15 trials. In the mixed context block, the 2400 ms duration was presented 10 times, whereas the other durations were presented 5 times (i.e., matching the number of presentations in the separate context blocks). Durations were presented in pseudo-random order. To ensure a sufficiently established context, the medium standard duration was not presented before the 5th or 9th trial in the separate/mixed blocks, respectively.

**Table 1:** Experimental design

	Part 1 (separate blocks)		Part 2 (mixed block)
Stimulus type realistic	Short 1066, 1600, 2400 ms	Long 2400, 3600, 5400 ms	Mixed 1066, 1600, 2400 ms/2400, 3600, 5400 ms
Stimulus type abstract	Short 1066, 1600, 2400 ms	Long 2400, 3600, 5400 ms	Mixed 1066, 1600, 2400 ms/2400, 3600, 5400 ms

Note that the order of presentation mode (separate vs. mixed) and stimulus type (abstract vs. realistic), as well as the assignment of contexts to spatial side (left or right) was counterbalanced across participants

Before the start of the experiment proper, participants performed practice trials that included performance-based feedback, until the task was sufficiently understood. No feedback was provided during experimental trials. Participants were instructed to refrain from chronometric counting.

### Statistical analysis

Data and analysis scripts can be found at https://osf.io/4bpag/. All analyses focussed on the medium standard duration of 2400 ms. Responses earlier than 0.5 s and later than 10 s were excluded from analysis (0.5% of the data). To test whether the reproduction of the standard duration was influenced by context, we fitted a linear mixed model (LMM) using the *lme4* package (Bates et al., 2015) and the *lmerTest* package (Kuznetsova et al., 2017), using Satterthwaite’s degrees of freedom method, in R (R Core Team, 2016), with the reproduced duration (expressed in seconds) as dependent variable. We included fixed factors for *context* (short vs. long, coded as − 0.5 and 0.5), *presentation mode* (separate vs. mixed, − 0.5 and 0.5) and *stimulus type* (abstract vs. realistic, − 0.5 and 0.5) and all first and second order interactions, and, based on our earlier work (Maaß et al., 2019), we included an interaction between *context* and the score on the word recall task (*RecallTask*)*.* Instead of coding word recall score as a binary variable as is common procedure, we utilized the higher precision that can be obtained by entering a numerical deviation from a cut-off score that is corrected for age, sex and education. The cut-off criteria were identical to the original version of this task in the CERAD test battery (Morris et al., 1989). The deviation from the corrected cut-off score was entered into the model as a continuous variable. In addition, a random intercept for subjects was added.

Moreover, we ran two additional models which focused on the explanatory power of the MoCA test. For these models we removed the RecallTask-based interaction, but included either an interaction between *context* and binary *MoCA* (score lower than 26 vs. higher than 25, coded as 0.5 and − 0.5 respectively, so that *MoCA* expressed the effect of scoring lower than the cut-off) or *context* and the *MoCA* memory subscore (median split on only the scores of the memory components of the *MoCA*, i.e., delayed recall, orientation and forward digit span; see Lam et al., 2013).

## Results

**Fig. 2:**
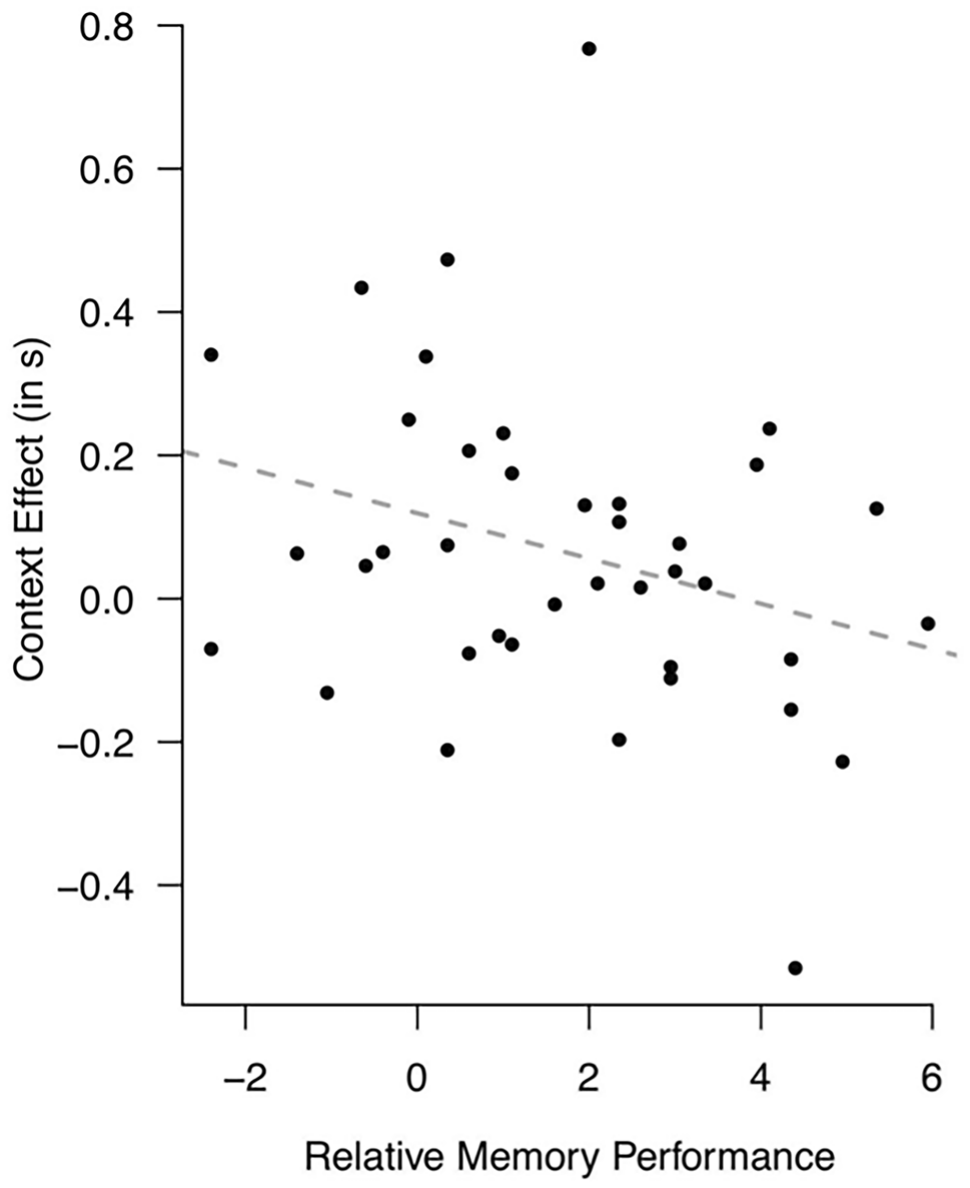
Context effects (defined as the difference between reproductions of the medium standard duration in the long and the short context) as a function of memory performance (defined as the recall task cut-off distance score), plotted per participant. The dashed line depicts the regression line

As expected, the base model contained a significant intercept (*β* = 2.22, SE = 0.06, *df* = 37.00, *t *= 38.41, *p* < 0.001) and a significant overall effect of *context* (*β* = 0.07, SE = 0.03, *df* = 1508.01, *t* = 2.52, *p* = 0.012), indicating that context indeed influences the reproduced duration for the medium standard duration. The interaction between *RecallTask* and *context* indicates that the recall task score modulates the context effect (*β* = − 0.03, SE = 0.01, *df* = 1508.00, *t* = − 2.54, *p* = 0.011): For each additional point on the recall task, the context effect is 30 ms smaller, supporting the hypothesis that the context effect is smaller for participants with better memory performance (Fig. 2).

The main effects of *stimulus type* and *presentation mode* indicate that realistic stimuli (*β* = 0.14, SE = 0.03, *df* = 1508.02, *t* = 5.42, *p* < 0.001) and mixed blocks (*β* = 0.05, SE = 0.03, *df* = 1508.01, *t* = 2.01, *p* = 0.044) increase the overall reproduced duration (i.e., independent of context). More importantly, where *presentation mode* did not modulate the context effect (*presentation mode* × *context:* *β* = − 0.05, SE = 0.05, *df* = 1508.02, *t* = − 0.89, *p* = 0.376), *stimulus type* did (*stimulus type* × *context:* *β* = 0.11, SE = 0.05, *df* = 1508.01, *t* = 2.04, *p* = 0.045), indicating that when realistic stimuli are presented, the difference between the two contexts is 110 ms larger than when abstract stimuli are presented. Neither the interaction between *presentation mode* and *stimulus type*, nor the three-way interaction between *presentation mode*, *stimulus type* and *context* reached significance (all: *t* < 0.89, *p* > 0.291). Model comparisons between this model and the models including higher order interactions indicated that the more complex models were not warranted (all: χ^2^ < 0.187, *df* = 1, *p* > 0.66).

**Fig. 3:**
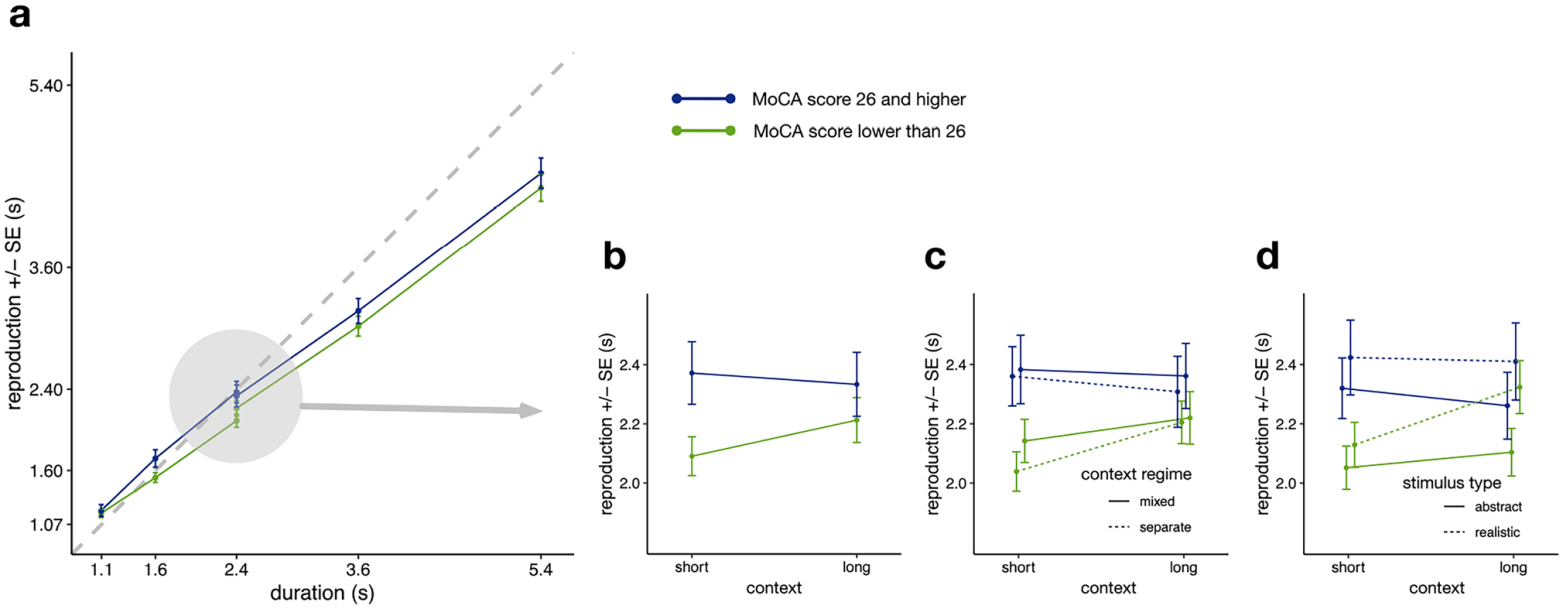
Effect of MoCA score on context effects. **a** Average interval reproductions as a function of MoCA scores. **b** Reproductions of the medium standard duration (i.e., 2400 ms) as a function of MoCA scores. **c** Reproductions of the medium standard duration as a function of MoCA scores and presentation type (i.e., whether contexts were presented in separate or mixed blocks). **d** Reproductions of the medium standard duration as a function of MoCA scores and stimulus type (abstract vs. realistic). Error bars represent standard errors of the mean with the within-participants Cousineau–Morey correction applied

The MoCA-based model contained a significant intercept (*β* = 2.25, SE = 0.06, *df* = 37.00, *t* = 37.99, *p* < 0.001). Interestingly, there is no overall effect of *context* (*β* = 0.04, SE = 0.03, *df* = 1508.01, *t* = 1.58, *p* = 0.114), yet the interaction between *MoCA* and *context* indicates that MoCA status modulates the context effect (*β* = 0.16, SE = 0.05, *df* = 1508.01, *t* = 2.99, *p* = 0.003), with participants failing the MoCA threshold showing a larger context effect. Specifically, participants failing the MoCA threshold showed a 160 ms larger reproduction difference between short and long contexts than participants passing the MoCA threshold (Fig. 3). The other main and interaction effects were qualitatively similar to those of the *RecallTask* model reported above. For both the short and the long context, slopes were smaller for the low scoring MoCA group (i.e., 0.67 and 0.68, respectively) than for the higher scoring MoCA group (0.79 and 0.76; cf. Fig. 3a).

The model including the MoCA memory subscore (the summation of the memory components of the MoCA) contained a significant intercept (*β* = 2.21, SE = 0.06, *df* = 37.00, *t* = 35.56, *p* < 0.001) and a significant main effect of *context* (*β* = 0.09, SE = 0.03, *df* = 1508.00, *t* = 3.37, *p* < 0.001). Again, an interaction with *context* indicates that the MoCA memory score modulates the context effect (*β* = 0.16, SE = 0.05, *df* = 1508.00, *t* = 2.98, *p* = 0.003). The other main and interaction effects were qualitatively similar to those of the *RecallTask* model reported above.

## Discussion

The perception of temporal intervals is highly influenced by the temporal context in which the physical interval is situated. In a time reproduction task, an interval of 2.4 s triggers a longer reproduction when it is presented within a set of even longer intervals than when it is presented together with shorter intervals (Maaß et al., 2019; Petzschner et al., 2015). Although the physical duration is the same, the context shapes its experience.

In the present study, we tested the influence of temporal contexts on time reproduction in older adults and assessed the influence of cognitive status on their temporal performance. On the basis of our previous study (Maaß et al., 2019), we reasoned that cognitive decline, especially regarding memory functions, coincides with a pronounced susceptibility to contextual information.

The results support the notion that individuals with lower memory scores exhibit stronger context effects than individuals with higher scores. Additionally, it demonstrates the generalizability of the observed effect as (1) they were also observed in a more naturalistic task setting, (2) remained present when the two earlier presented contexts were mixed in a final block, and (3) the durations in this experiment were about twice as long (but still in the range of seconds) as the durations used in Maaß et al. (2019). Moreover, the results were independent of the method by which memory or more general cognitive functioning was assessed, as the same pattern of results was observed both when memory functioning was assessed by a delayed word recall task modelled after the CERAD test battery (Morris et al., 1989), and when using the scores on the Montreal Cognitive Assessment (MoCA; Nasreddine et al., 2005). Both of these measures confirmed an influence of the temporal context.

### Temporal context effects as behavioral marker for cognitive decline in advanced age

Our results solidify and extend the observation that memory deficits in advanced age are linked to a more pronounced effect of temporal contexts on the reproduction of durations. Older adults with beginning signs of impaired mnemonic capacities were more influenced by the temporal context in which a specific duration was presented. This finding points to the potential value of timing tasks for the clinical diagnostics of cognitive decline (El Haj & Kapogiannis, 2016; Maaß et al., 2019). Timing abilities are known to deteriorate with increasing age (Espinosa-Fernández et al., 2003; Gooch et al., 2009; Mioni et al., 2019), and especially pathological cognitive decline coincides with specific impairments in the perception of temporal intervals (Caselli et al., 2009; El Haj et al., 2013; Rueda & Schmitter-Edgecombe, 2009).

In a recent study we have shown that patients with amnestic mild cognitive impairment (aMCI) exhibit stronger temporal context effects than healthy, age-matched controls (Maaß et al., 2019). Importantly, we ruled out the possibility that these effects were driven by changes in clock variability, as temporal performance in a 1-s production task (Maaß & van Rijn, 2018) did not explain the observed context effects. We therefore assumed that the reason for the increased effect of temporal context might consist in a stronger weighting of prior experiences. Interestingly, an increased reliance on contextual temporal information was also observed in the subgroup of the healthy participants that performed below average on a memory test, demonstrating the sensitivity of temporal context effects to beginning deficits in memory functions. Corroborating these findings, the present study shows that in older participants, temporal context effects are associated with cognitive status in a sample that was not pre-diagnosed with any memory deficits.

In addition to the correlation between context effects and memory functioning (assessed by a delayed word recall task), we also found that the Montreal Cognitive Assessment survey (MoCA; Nasreddine et al., 2005) is associated with a stronger influence of temporal contexts. This further confirms the usefulness for clinical diagnostics, as the MoCA is a widely accepted screening tool for general cognitive functioning and frequently used to identify patients at risk for pathological cognitive decline. The assessment of context effects via time reproduction tasks has great potential as a diagnostic measure for memory dysfunction in older age, because these tasks are not affected by repeated testing sessions. In other words, while conventional tests for memory dysfunctions (e.g., learning and retrieval of word lists) show clear effects of repeated assessments (Dikmen et al., 1999), and the construction of parallel test versions is costly and only possible to a certain degree, the assessment of temporal context effects is arguably less affected by repeated testing (especially when test sessions are separated by more than a month), which would enable an unlimited series of consecutive applications. Therefore, time reproduction tasks represent a valuable diagnostic tool to assess the progress or decline of memory functions, for example, to quantify the effectiveness of clinical treatments.

### The benefit of realistic stimuli

Our results further show that context effects were larger for the realistic task version, in which the to-be-timed durations were embedded in a more naturalistic context rather than being demarcated by abstract, meaningless shapes (cf. Fig. 1). This finding corroborates the idea that a semantic embedding of stimuli increases the tendency to process regularities (Peelen & Kastner, 2014; Thanopoulos et al., 2018), and hence facilitates the detection of different temporal contexts. In our design, short and long temporal contexts were associated with either the left or the right spatial side. If the processing of these regularities (i.e., the mapping between spatial side and temporal context) is easier in the realistic as compared to the abstract task version, then we would expect that, in the realistic task version, the medium standard duration is more affected by the two distinct contexts. This hypothesis was confirmed. Thus, in contrast to abstract stimuli like visual shapes, which do not contain inherent meaning, semantically embedded stimuli seem to prompt the tendency to process temporal regularities and to take them into account for time reproductions. It should be noted that the durations used in this study are not necessarily plausible for the specific events (i.e., a window is usually not illuminated for only a few seconds). This further demonstrates that the effect is driven by the realistic context, which induces a general tendency to process temporal regularities, rather than by a realistic combination of events and durations. An open question for future studies is whether temporal context effects can be further increased by a plausible combination of events and durations.

The present results, however, do not reveal a three-way interaction between the temporal context (short vs. long), stimulus type (abstract vs. realistic) and presentation mode (separate vs. mixed), indicating that the semantic embedding of stimuli had a similar impact on context effects during separate and mixed presentation. This contradicts the idea that the effect of semantic embedding is more pronounced when short and long contexts are demarcated only by spatial aspects of the environment. Instead, it shows that the semantic embedding of stimuli increases the influence of temporal contexts both in separate and mixed blocks. In future studies, the assumption of an increased temporal generalization in realistic environments can be further tested by assessing context effects in separate blocks, with the durations being demarcated by a single vs. various parts of realistic vs. abstract environments.

In an attempt to increase the ecological validity of timing experiments, many researchers have argued for the use of more realistic stimuli (Boltz, 2005; Matthews & Meck, 2014; Riemer et al., 2018; Schlichting et al., 2018) and for the implementation of more natural paradigms (Matthews & Meck, 2014; Tobin et al., 2010; van Rijn, 2014). In support of this claim, the present study highlights the importance of ecological validity by showing that a specific perceptual distortion of temporal information is more pronounced for naturalistic as compared to abstract stimuli. The complexities of and interactions between temporal characteristics of naturalistic stimuli in the real world might not be adequately represented in experiments relying only on abstract stimuli.

### Context effects in separate vs. mixed blocks

In the present study, we investigated context effects in separate and mixed blocks (i.e., when left- and right-sided stimuli are associated with different ranges of durations), and we did not find a difference between these presentation modes. This contrasts with a study by Roach et al. (2017), who found that context effects in a mixed block are relatively hard to establish and depend—under normal conditions—on a considerable number of trials. Several aspects of the current study might explain this inconsistency. First, the group we tested here consisted of older adults with beginning signs of cognitive deficits, while Roach et al. (2017) investigated young, healthy individuals, and it has been shown that aging and cognitive deficits coincide with a higher susceptibility to context effects (Maaß et al., 2019; Shapiro & Levy-Gigi, 2016). Second, the order of presentation modes was fixed in our experimental design, so that each participant performed first the blocked presentation of both separate temporal contexts and then the mixed presentation. This procedure might have enhanced the salience of the difference between the two contexts. An important next step for future research therefore consists in the study of temporal context effects in mixed blocks *without* previous adaptation to the contexts in separate blocks.

### The neuronal origin of temporal context effects

Establishing temporal context effects as a behavioral marker for age-related cognitive decline poses the question about the neuronal origin of these effects (e.g., whether they occur at an early perceptual processing level or at a later, decisional stage). Age-related cognitive deficits have often been linked to disturbances in sensory processing (Kuehn et al., 2018; Moran et al., 2014). For example, based on reports of a reduced mismatch negativity in older adults (Cheng et al., 2013), Moran et al. (2014) argued that, for the sake of higher robustness, the aging brain favors less complex models of the environment, ultimately giving rise to a generalization of sensory input and an increased reliance on prior information. In accordance with this view, a recent study provided support for the idea that also temporal context effects occur at an early level of perceptual processing (Zimmermann & Cicchini, 2020). The authors could show that introducing sensory uncertainty for the to-be-timed stimulus led to a more pronounced context effect, a finding that cannot be explained on the basis of decisional processes alone.

## Conclusions

Together with our previous study (Maaß et al., 2019), the present results suggest that temporal context effects reveal valuable information about the cognitive status of older adults. Furthermore, our results show that context effects can be observed in a mixed presentation mode including two distinct temporal contexts within the same block. In the future, respective timing tasks might prove a useful diagnostic tool for pathological cognitive decline at an early, pre-clinical stage, and therefore it is important to optimize the experimental conditions under which temporal context effects can be measured. The present study demonstrates an advantage for stimuli which are semantically embedded within a naturalistic context, thereby suggesting a simple way to improve on previous paradigms to probe temporal context effects.

## Data Availability

The datasets generated during and/or analysed during the current study are available at the Open Science Framework (OSF): https://osf.io/4bpag/
